# One-Story versus Many-Story Methods

**Published:** 1919-05-17

**Authors:** 


### HOSPITAL CONSTRUCTION: THE NEW ERA.

### VII. One-Story Versus Many-Story Methods.

The question whether it is better to build a hospital on the one-story principle or in two or more floors one above the other is not easily answered in the abstract. Circumstances alter cases in hospitals as in other matters, and it often happens that circumstances—or one single circumstance—will settle the matter without dispute. The size of an available site will, for example, very often put an absolute veto on the bungalow system. On the other hand the urgency of time-pressure may in emergency cases make the one-story building an absolute necessity; because if a large plot of ground is at disposal and if a large gang of workmen can be secured, it is possible, by the dispersal of their operations over a wide space, to get a comparatively large building erected in an incredibly short time. Again, if emergency materials are being used it is probable or possible that wall construction of a light character can be resorted to which is quite strong enough for a low building but quite unsuited to a high one.

But there remains the case of a permanent hospital on a large site in which no hampering conditions of space or time tie the designer down to either method. Which way should the choice go? Shall we get the better, the more convenient building by the one-story or the several-story method? The advocate of the bungalow treatment will urge first of all that the avoidance of stairs and lifts saves cost in building and labour in locomotion. He will, secondly, point out that as the various blocks and units will all be low, they will not obstruct the passage of air and light to one another—as in a high building—and that the ward blocks can therefore be placed nearer to one another than is possible or desirable where twenty-five or forty feet is adopted as the height of the structure. He will probably bring forward as his third argument that low buildings do not involve the use of such thick walls as are necessary in tall ones, and that, consequently, there will actually be less material required per cubic foot of structure than can ever be the case where height necessitates strength of construction. To this he will add as a fourth reason a comment on the desirability of treating all scientific and hygienic buildings as, in a sense, temporary. He will point out that medical and surgical science is never stagnant, that all hospitals become in a comparatively short space of time obsolete, and that the construction of low and slight buildings contributes very conveniently to the possibility of alterations, whether it be experimental alteration of a single department or the gradual remodelling of an entire institution. His last shot will probably be the suggestion that the piling of ward upon ward is really a form of overcrowding. It is the allotting of more persons to the acre; and it leads, he will say, to greater contamination of air. Putting room over room renders possible the communication of diseased air from a lower ward to an upper, and in any case facilitates the disturbance of the patients by noises from above or from below.

All these arguments are fairly sound. But nearly all of them can be met by counter arguments. Perhaps the last of them is in some ways the strongest. It is quite true that a tall building packed tightly upon a restricted space does offer a tendency for the accumulation of used-up and contaminated air. It will happen at times that even in a building where the wards themselves are kept reasonably fresh by the use of good window ventilation, the stairs and corridors get, through excess of heating and neglect of window opening, stuffy, and perhaps unwholesome. To speak of placing too many patients in the acre is misleading. There is no magic healing-power in the tenancy of so much soil per invalid. It is, of course, the air occupation that matters, but it is to be admitted that there is at least some argument here for the one-story enthusiasts; for the one-story building does escape from the burden of effete used-up air conveyed by heating power from the lower to the upper stories.

The obstruction of air and light between ward and ward which is liable to occur in a high building should be counteracted by so planning the parts in regard to aspect that the sun is given an opportunity of visiting every ward for a good part of the day, and that the wind is not shut out from any nook or corner. This latter is an important point; there should, for example, never be in any hospital any courts or areas enclosed on all four sides.

When we come to consider the question of economy of materials, the case for the one-story advocates may be found to be not quite so strong as it at first appears. The one-story wards require each to have its own foundation and its own roof instead of sharing roof and foundation with two other wards, and what such buildings save in staircase space they lose in extra corridor, so that, except for the argument as to ease of alteration, there is not much economy of building material effected.

But the point at which the one-story theory most conspicuously breaks down is the point of practical convenience. The absence of stairs and lifts is not a saving in locomotion—quite otherwise. A fairly large institution built on the bungalow plan involves the staff in journeys of immense length. The conveyance, for example, of meals to the patients, and of patients to an operating-theatre or to a special treatment quarter, is rendered vastly more inconvenient in a large one-story building than in one of several floors with a good lift. Even members of the household staff who do not necessarily use the lift are better off in the building with stairs; for the change of exercise in walking up one or two flights may be less fatiguing to a healthy person than a long corridor.

In conclusion, it may probably be said that in a

☞ The previous articles appeared on January 25, February 8, March 1, 15 and 29, and April 19, p. 61.

**The New Era**—(*continued*).

small hospital—a hundred beds or less—it is preferable to place all the wards on one floor. This need not necessarily be the ground floor, for it may happen to be convenient to place various administrative or other non-ward departments under the spaces occupied by the wards. But care must be exercised in arranging this. One would not, for example, place an out-patient department beneath a ward nor any department productive of noise or annoyance, such as a kitchen or an electric-light plant. It is more usual, no doubt, to place the wards on the ground floor more or less radiating from a central administrative block which is raised to the height of as many stories as convenience dictates, and the arrangement is not a bad one.

But where the hospital contains hundreds of beds it must almost inevitably make for convenience to arrange the wards over one another on two or more floors well served by convenient staircases and a lift or lifts of sufficient size. Ease of administration, speed of service, economy of heating, simplicity of plumbing and general utility all conspire on the side of a reasonable adoption of the upstairs and downstairs type. There is one further condition worth consideration, the hill-side site. A hill sloping northward—always a bad site for a hospital—needs special care lest buildings which on the flat would not overshadow one another should increase the disabilities of the aspect. The southward inclination, on the other hand, leaves the architect exceptionally free and may tempt him to emphasise the advantages of the gradient by a judicious mixture of high buildings and low. A northward slope is fatal to a small site, as it defies expansion as well as forbidding undue height.

